# Nap polysomnography in infants with laryngomalacia as a tool to predict treatment strategy

**DOI:** 10.1007/s00405-024-08623-y

**Published:** 2024-04-04

**Authors:** Mariem Lajili, Natacha Teissier, Benjamin Dudoignon, Charlotte Benoit, Sophie Bellanger, Laureline Kahn, Thierry Van Den Abbeele, Christophe Delclaux, Plamen Bokov

**Affiliations:** 1 Service de Physiologie Pédiatrique-Centre du Sommeil, AP-HP, Hôpital Robert Debré, 75019 Paris, France; 2 Service d’Oto-Rhino-Laryngologie, AP-HP, Hôpital Robert Debré, Université de Paris-Cité, 75019 Paris, France; 3 Service de Physiologie Pédiatrique-Centre du Sommeil, INSERM NeuroDiderot, AP-HP, Hôpital Robert Debré, Université de Paris-Cité, 48, Boulevard Sérurier, 75019 Paris, France

**Keywords:** Sleep-disordered breathing, Supraglottoplasty, Polysomnography

## Abstract

**Purpose:**

This study aimed to investigate the role of nap polysomnography (NPSG) in predicting treatment strategies for infants with moderate to severe laryngomalacia and to explore the association between obstructive sleep apnea (OSA) severity, weight gain, and laryngomalacia severity.

**Methods:**

A retrospective analysis was conducted on infants diagnosed with moderate to severe laryngomalacia who underwent NPSG between January 2019 and June 2023. Clinical variables, NPSG parameters, and treatment decisions were collected. Weight gain rate and its correlation with NPSG indices were assessed. Logistic regression analyses were performed to predict treatment strategies based on NPSG findings.

**Results:**

Of the 39 infants included (median age: 3.3 months), 77% exhibited OSA, with 69% having moderate to severe OSA [apnea–hypopnea index (AHI) > 5/h]. Weight gain rate correlated negatively with indices of OSA severity, including the hypopnea index (HI) and the AHI. In a multiple logistic regression analysis incorporating the severity of OSA (AHI), weight gain rate, and laryngomalacia severity, only AHI predicted the decision for surgical or non-invasive ventilation treatment (OR = 2.1, CI_95_ [1.6; 2.8], *p* ≤ 10^–4^). The weight gain rate was predicted (*r*^2^ = 0.28) by the AHI and the presence of retractions of auxiliary inspiratory muscles.

**Conclusion:**

This study underscores the importance of NPSG in assessing infants with moderate to severe laryngomalacia. The AHI from NPSG emerged as a potential predictor for treatment decisions and weight gain rate, emphasizing its clinical relevance. These findings advocate incorporating NPSG into the diagnostic and management process for infants with laryngomalacia.

## Introduction

Laryngomalacia is the most prevalent congenital laryngeal anomaly in infants and poses a significant challenge in clinical management due to its diverse and often elusive presentation [1]. Laryngomalacia is defined as the collapse of the supraglottic structures during inspiration-producing stridor. Signs of severity are poor weight gain (probably the most contributing element) [2], permanent and severe intercostal or xiphoid retraction, and episodes of respiratory distress, cyanosis, or apnea. Moreover, recent studies have highlighted the potential association between laryngomalacia and sleep-disordered breathing, particularly obstructive sleep apnea (OSA) [3, 4]. Understanding the intricate relationship between OSA, poor weight gain, and the severity of laryngomalacia holds considerable clinical implications, potentially guiding treatment strategies for affected infants.

The role of polysomnography (PSG) in the diagnostic work-up and decision-making is not clear. The International Pediatric ORL Group (IPOG) laryngomalacia consensus recommendations of 2016 [5] advocate considering PSG or home oximetry monitoring if significant apnea is present. The authors also suggest that the presence of OSA could potentially sway decisions toward surgical interventions [5]. Indications for supraglottoplasty in the case of laryngomalacia include severe stridor with compromised airways, feeding difficulties, failure-to-thrive, and OSA [1]. Previous studies have shown that infants with failure-to-thrive related to laryngomalacia do grow after supraglottoplasty [6, 7].

This study aims to delve into the role of nap polysomnography (NPSG) as a predictive tool for determining appropriate medical (non-invasive ventilation) or surgical treatment strategies for infants diagnosed with moderate to severe laryngomalacia. NPSG involves a comprehensive sleep assessment during spontaneous daytime sleep, allowing for a detailed evaluation of respiratory events, sleep patterns, and oxygen saturation levels.

In addition to assessing NPSG’s predictive value, this study will explore the correlation between the severity of OSA and the extent of laryngomalacia, as well as the connection between OSA and inadequate weight gain, a more objective indicator of laryngomalacia’s clinical severity.

## Methods

We conducted a retrospective analysis of medical records encompassing infants who underwent NPSG and were diagnosed with laryngomalacia, confirmed through direct laryngoscopy. The study spanned from January 2019 to June 2023. These infants were suspected of having OSA based on symptoms such as stridor accompanied by witnessed apnea or positive apnea history, or retraction of auxiliary inspiratory muscles during wakefulness. Infants with cardiorespiratory or neuromuscular conditions, dysmorphic features, significant craniofacial abnormalities or chromosomal syndromes were excluded. Additional exclusion criteria comprised unsuccessful NPSG due to insufficient sleep duration (less than 2 sleep cycles recorded) and acute respiratory infections during NPSG. Furthermore, infants with a history of current or prior surgical or non-invasive ventilation (NIV) treatment for OSA were also excluded. Ethical approval was obtained from the local Ethics Committee (CEER-0012-2017), and the data collection was registered with the French regulatory agency (CNIL). The subjects and their parents were informed of the collection of their prospective data for research purposes, and they could request to be exempted from this study as per French law (non-interventional research). The study adhered to the STROBE criteria for cohort studies.

### Clinical examination and retrieval of clinical variables

Clinical examination and data retrieval encompassed the following variables: sex, gestational term, disease severity, weight, and age at the initial ear, nose, and throat (ENT) office visit. Laryngomalacia diagnosis was based on systematic flexible laryngoscopy performed in an office setting, involving nasal airway access to confirm laryngomalacia and exclude other potential causes of supraglottic obstruction. Laryngomalacia severity was scored according to IPOG consensus recommendations [5]. Briefly, moderate laryngomalacia is considered in the presence of clinical signs as cough, choking, regurgitation or feeding difficulties, and severe laryngomalacia if one of the following is reported: witnessed apnea or cyanosis, failure to thrive, clinical signs of cor-pulmonale.

Additional variables included the type of laryngomalacia [1], comorbidities, presence of apnea during clinical examination or positive apnea history, cyanotic episodes, retraction of auxiliary inspiratory muscles, and treatment status. Weight and age were collected on the day of NPSG and at a subsequent ENT office visit for reassessment. Failure-to-thrive was defined as a weight below the 5th percentile for chronological age at the time of NPSG [8].

### Weight gain rate

**Fig. 1:**
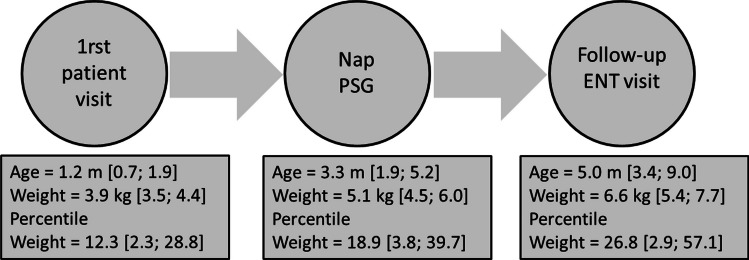
Timeline (age in months) from the first clinical visit to follow-up. PSG is polysomnography

The weight gain rate was calculated by assessing the percentile change in weight between the first pre-NPSG evaluation and a subsequent post-NPSG evaluation, divided by the time interval between the two assessments (Fig. 1).

### In-laboratory polysomnography

NPSG studies were conducted during spontaneous morning sleep. A minimum of two sleep cycles was set as the required duration. An Alice 6 LDx polysomnography system (Philips, Murrysville, PA) recorded the following parameters: chest and abdominal wall motion using respiratory inductance plethysmography, heart rate by electrocardiogram, arterial oxygen saturation (SpO_2_) by pulse oximetry, transcutaneous PCO_2_, airflow using a three-pronged thermistor, nasal pressure by a pressure transducer, electroencephalographic leads (C3/A2, C4/A1, F3A2, F4A1, O1/A2, O2/A1), left and right electrooculograms, and submental electromyogram. Study participants were also recorded using an infrared video camera. Respiratory events, including apneas and hypopneas, were scored based on the American Academy of Sleep Medicine (AASM) scoring manual version 2.4 [9] by experienced pediatric sleep physicians. Hypopnea was scored if there was a 30% or more reduction of the pressure transducer signal from the pre-event baseline for at least two breaths, and it was associated with a drop in oxygen saturation of at least 3% or an arousal. OSA was defined by an obstructive apnea–hypopnea index (OAHI) ≥ 2/h, moderate to severe OSA by an AHI > 5/h [10]. Sleep stages were categorized as R [equivalent to rapid eye movement (REM) sleep] or N (equivalent to NREM sleep) for infants under 2 months at the time of the polysomnography as recommended [11].

### Statistical analyses

Results were expressed as medians [25th–75th percentiles]. Comparisons of continuous variables between groups were performed using the *t*-test or the Wilcoxon test when data violated assumptions of normality. When appropriate, categorical variables were compared using the chi-square or Fisher’s exact tests. Normality was checked using the Shapiro–Wilk test. Correlations were evaluated using Pearson’s correlation coefficient. Additional statistical analyses are described in the text. All *p*-values reported are two-tailed, with statistical significance set at < 0.05. All statistical analyses were performed with R software version 4.1.0.

### Sample size

A rule of thumb for logistic regression is to include at least 5–9 events per predictor [12]. In a study by Verkest and colleagues [3], 64% (28 over 44) of the infants were treated by supraglottoplasty or NIV; in another study by Weinstein and colleagues [13] including 23 infants with moderate to severe laryngomalacia, 70% of them were treated by supraglottoplasty. Thus, we calculated that at a minimum sample size of 3 × 5/0.7, 22 infants would be sufficient for a multiple regression analysis with a maximum of 3 predictors.

## Results

### Clinical characteristics

We included 39 subjects in the analysis. The timeline from the first office visit to the follow-up is shown in Fig. 1. All infants were diagnosed with moderate to severe laryngomalacia and were receiving acid suppression therapy as per recommended guidelines [5].

The clinical characteristics of the infants are given in Table 1. Sleep study variables are shown in Table 2. A diagnosis of OSA was established in thirty out of thirty-nine infants (77%, 95% confidence interval, CI_95_ [61; 89]), and moderate to severe OSA was diagnosed in 27 (69%, CI_95_ [52; 83]) infants.

**Table 1:** Clinical characteristics of the study infants (*n* = 39)

Characteristics	Variable	*n* (%)
Anthropological	Sex, male	23 (59)
	Term children	31 (79)
	Age at NPSG, months	3.3 [1.9; 5.2]
	Weight at NPSG, kg	5.1 [4.5; 6.0]
	Weight percentile at NPSG	18.9 [3.8; 39.7]
Clinical	Stridor, Permanent	15 (38)
	Failure to thrive	10 (26)
	Feeding problems	12 (31)
	Retractions	26 (67)
	Witnessed apnea	21 (54)
	Cyanosis episodes	4 (10)
Flexible laryngoscope exam	Laryngomalacia type, I/II/mixed	2/26/6
	Severity, moderate/severe^a^	11/27
First line treatment	Supraglottoplasty	12 (31)
	NIV	1 (3)

*NPSG* nap polysomnography, *NIV* non-invasive ventilation

^a^ Information for the severity of laryngomalacia was unavailable for one patient

**Table 2:** Nap polysomnography findings

Variable	Median [25th; 75th percentile]
TST, min	127 [111; 151]
NREM, %	55 [48; 63]
REM, %	45 [37; 52]
Arousal index, /h	13.5 [10.2; 16.2]
AHI, /h	9.9 [4.6; 22.2]
OAHI, /h	7.5 [2.7; 19.3]
OAI, /h	2.1 [0.3; 6.3]
HI, /h	4.4 [1.4; 12.3]
CAI, /h	1.0 [0.4; 2.4]
Obstructive apnea duration, mean (s)	6.2 [3.9; 7.0]
Hypopnea duration, mean (s)	8.3 [6.9; 10.1]
ODI, /h	6.5 [3.3; 16.9]
SpO_2_ min, %	91 [87; 93]
*T* < 90%, %	0 [0; 0.3]
PtcCO_2_ max, mmHg	46 [42; 48]
Stridor, % TST	30 [4; 76]

*TST* total sleep time, *NREM* non-rapid eye movement sleep, *REM* rapid eye movement sleep, *AHI* apnea–hypopnea index, *OAHI* obstructive apnea–hypopnea index, *OAI* obstructive apnea index, *HI* hypopnea index, *CAI* central apnea index, *ODI* desaturation index, *T* *<* *90%* time spent under a SpO_2_ of 90%, *PtcCO*_*2*_ transcutaneous PCO_2_

The duration of flow limitation associated with hypopneas was 43.6 [15.9; 114.6] s/h compared to 12.0 [1.6; 44.9] s/h associated with obstructive apneas (*p* = 0.004).

### Bivariate analyses

We observed a median weight gain rate of 0.04 percentile/day [0.00; 0.23], min = − 0.46, max = 0.66. Weight gain rate was negatively correlated with AHI (*r* = − 0.46, *p* = 0.007), OAHI (*r* = − 0.45, *p* = 0.009), HI (*r* = − 0.45, *p* = 0.008), and oxygen desaturation index (IDO, *r* = − 0.41, *p* = 0.019). The correlation was not significant with OAI (*p* = 0.123). Weight gain rate (measured in percentile.day^−1^) in children with retractions of auxiliary inspiratory muscles was 0.02 ± 0.21 vs. 0.25 ± 0.22 in those without (*p* = 0.012).

The OAI, central AI, and oxygen desaturation index were not significantly different between infants with moderate or severe laryngomalacia, while AHI, OAHI, and HI were (Table 3).

**Table 3:** Sleep study parameters according to the severity of laryngomalacia

Characteristics	Moderate^a^ laryngomalacia *N* = 11	Severe laryngomalacia *N* = 27	*p* value
Sleep study parameters			
AHI, /h	4.8 [3.0; 10.2]	16.6 [5.7; 40.8]	0.031
OAI, /h	0.5 [0.1; 4.2]	2.5 [0.6; 16.7]	0.179
CAI, /h	0.6 [0.5; 2.5]	1.4 [0.2; 2.2]	0.859
OAHI, /h	4.2 [1.5; 7.7]	16.3 [3.5; 36.3]	0.041
HI, /h	2.2 [0.6; 4.8]	6.8 [3.4; 20.2]	0.008
ODI, /h	4.5 [2.0; 7.9]	8.8 [4.6; 30.0]	0.055

*AHI* apnea–hypopnea index, *OAHI* obstructive apnea–hypopnea index, *OAI* obstructive apnea index, *HI* hypopnea index, *CAI* central apnea index, *ODI* desaturation index

^a^Information for severity was unavailable for one patient

AHI in infants with witnessed apnea or a positive history of apnea was not different from the AHI in those without; 9.8/h [4.2; 21.8] vs. 10.2/h [4.7; 20.1], *p* = 0.854, respectively.

### Multivariate analysis of weight gain rate

A multiple regression analysis, with weight gain rate as the dependent variable and signs of retraction and the AHI as predictors, showed that the two variables were independently and negatively related to the weight gain rate (Table 4). Similar results were observed with the HI and the OAHI.

**Table 4:** Multiple linear regression model to predict poor weight gain rate using the presence of retractions and one polysomnographic index (HI, AHI, or OAHI)

	*B*	SE_B_	*β*	*p* value
Model	Adjusted *r*^2^ = 0.28			
Retraction	− 0.178	0.079	− 0.76	0.031
AHI	− 0.004	0.002	− 0.34	0.028

*B* unstandardized beta, *SE*_*B*_ standard error of unstandardized beta, *β* standardized beta, *AHI* apnea–hypopnea index

### Prediction of treatment strategy

**Fig. 2:**
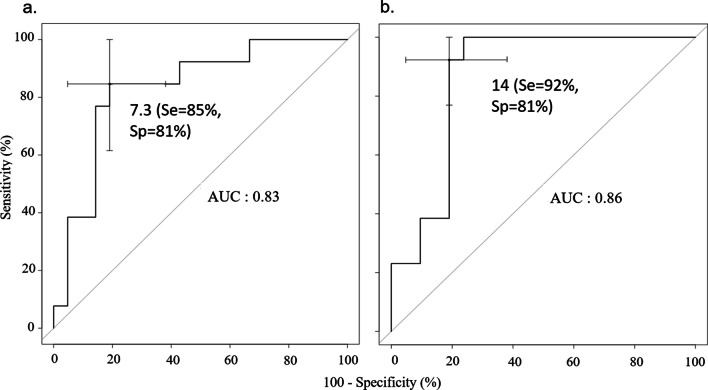
Receiver operating characteristic curve for prediction of treatment (surgery or NIV) **a** by HI and **b** by AHI. The thresholds producing the best performances (minimal distance to the upper left corner) are given along with the corresponding Se and Sp. AUC is the area under the curve. NIV is non-invasive ventilation. HI and AHI are the hypopnea and the apnea-hypopnea indexes, respectively

We constructed a composite variable that took into account the decision to treat an infant presenting with laryngomalacia (by surgery or by NIV). The decision to treat was predicted by the severity of OSA as measured by the AHI or HI. In Fig. 2, we present the receiver operating characteristic curve of the AHI and HI as predictors of the decision to treat. The threshold value of 14/h for the AHI outperformed the threshold value of the HI (7.3/h) as the distance to the upper left corner was shorter (20.5 vs. 24.5). In a multiple logistic regression (Table 5) with the decision to treat it as the dependant variable and the AHI > 14/h, and negative weight gain and severe laryngomalacia as predictors, only the AHI > 14/h was independently associated with the decision to treat (OR = 2.1, [1.6; 2.8], *p* ≤ 10^–4^).

**Table 5:** Multiple logistic regression model to predict treatment strategy

	B	SE_B_	Odds ratio	Odds ratio, CI_95_	*p* value
Traits model	McFadden’s *R*^2^ = 0.53				
AHI > 14/h	0.74	0.15	2.1	[1.6; 2.8]	< 10^–4^
Weight gain rate < 0	0.04	0.16	1.0	[0.8; 1.4]	0.810
Severe laryngomalacia	− 0.19	0.16	0.8	[0.6; 1.1]	0.204

*B* unstandardized beta, *SE*_*B*_ standard error of unstandardized beta, *HI* hypopnea index

## Discussion

The main finding of our retrospective study strongly suggests that OSA significantly influences the decision-making process when treating infants with moderate to severe laryngomalacia.

Our study cohort shared similar age and clinical characteristics with other research groups [3, 4, 13]. However, a distinct aspect of our approach is that we conducted sleep studies exclusively on infants with laryngomalacia who displayed retractions of auxiliary inspiratory muscles or had a positive history of apnea/witnessed apnea during office ENT visits. The prevalence of OSA in our study was 77% [61; 89], which closely mirrors the rates observed by others (77–87%) [3, 14] using the same OSA definition.

Notably, we observed more pronounced AHI values in infants with severe laryngomalacia than in those with moderate cases. Interestingly, this result might seem to contradict the findings of Weinstein and colleagues [13], who found no correlation between the AHI and the severity score of laryngomalacia. In our analysis, this distinction was driven by the HI, while the obstructive and central events were not significantly different (Table 3).

Another aspect where the mentioned study differs is the method for assessing the severity of laryngomalacia. In our case, we employed the IPOG recommendations. In contrast, Weinstein and colleagues [13] utilized the grading score proposed by Sivan and colleagues [15] that considers historical data, physical examination results, and laryngoscopic observations.

Furthermore, we observed that the AHI and the presence of retractions observed during clinical examination served as significant predictors of poor weight gain. These findings underscore the potential role of OSA in influencing growth outcomes and emphasize the importance of both objective polysomnographic assessment and comprehensive clinical evaluations when making management decisions for infants with laryngomalacia. Although there is currently no existing literature on the clinical differences between obstructive apnea and hypopnea in infants, one plausible explanation for our findings lies in the longer duration of flow limitation associated with hypopneas. This observed association could be attributed to the fact that sleep-related flow limitation and increased upper airway resistance are connected to heightened work of breathing in symptomatic children with OSA [16], providing a potential explanation for the strong associations between the HI and clinical outcomes in infants with laryngomalacia. Poor weight gain has been associated with OSA in infants [17]. While the precise mechanisms underlying this growth limitation are not fully elucidated, factors may include an increased metabolic rate during sleep, particularly during the REM stage. In a study by Marcus and colleagues [18], oxygen consumption during sleep decreased after adenoidectomy and tonsillectomy in children with OSA. These findings suggest that post-treatment weight gain could potentially stem from reduced energy expenditure during waking, but also sleep, with the pre-treatment increase in energy expenditure with retractions of auxiliary inspiratory muscles during waking and respiratory effort during sleep consuming oxygen required for growth. Interestingly, no significant correlation was found between the severity of OSA, as measured by the AHI, and sleep-related energy expenditure [18, 19].

Nevertheless, it is important to acknowledge some limitations of our study. Its retrospective nature and potential missing clinical data are notable drawbacks. Patient selection could also have been influenced by parental demand for polysomnography. Another potential limitation pertains to the daytime sleep recorded by polysomnography, which is considered a less validated approach than the gold standard overnight polysomnography. Nonetheless, a recent study by Singh and colleagues [20] demonstrated equivalent polysomnographic sleep parameters and treatment prescriptions, regardless of whether the polysomnography was conducted during daytime or nighttime in infants up to 6 months of age. One strength of our study lies in the homogeneity of our population, which exclusively included infants with isolated laryngomalacia, thus minimizing potential influences on weight gain rate and disease severity from syndromic cases.

Our study advocates for incorporating polysomnography as an additional tool for predicting clinical severity, particularly concerning poor weight gain, in infants with moderate to severe laryngomalacia. Importantly, we emphasize the value of polysomnography irrespective of the history of apnea. The AHI has emerged as a potentially valuable paraclinical marker for severity, offering additional guidance for treatment decisions. However, further research is necessary to validate the clinical utility of an AHI threshold of 14/h.

By highlighting the significance of NPSG in evaluating and managing infants with laryngomalacia, this research aims to foster collaboration among pediatricians, otolaryngologists, and sleep specialists. This interdisciplinary approach holds promise for enhancing the comprehensive care of these infants and has the potential to pave the way for improved long-term outcomes.

## Conclusion

In our cohort, the respiratory indices derived from NPSG proved potential to become predictive indicators for the requirement of supraglottoplasty or NIV in children afflicted by moderate to severe laryngomalacia. Furthermore, these specific parameters, with particular emphasis on the AHI, correlated with the weight gain rate. We identified a threshold value for the AHI that played a role in determining the treatment course. This finding, however, requires further validation through prospective investigation in a distinct cohort.

## Data Availability

The data presented in this study are available on request from the corresponding author.
